# How current perspectives on algorithmic thinking can be applied to students’ engagement in algorithmatizing tasks

**DOI:** 10.1007/s13394-023-00462-0

**Published:** 2023-06-13

**Authors:** Timothy H. Lehmann

**Affiliations:** School of Teacher Education and Leadership, Faculty of Education, Queensland University of Technology, E Block, Level 3, Victoria Park Road, Kelvin Grove, QLD 4059 Australia

**Keywords:** Algorithmic thinking, Algorithm, Graph theory, Maximum flow, Discrete mathematics

## Abstract

The aim of this study is to examine how algorithmatizing tasks engage mathematics students in algorithmic thinking. Structured, task-based interviews were conducted with eight Year 12 students as they completed a sequence of algorithmatizing tasks involving maximum flow problems. A deductive-inductive analytical process was used to first classify students’ mathematical behavior according to four cognitive skills of algorithmic thinking (decomposition, abstraction, algorithmization, and debugging) and then develop sets of subskills to describe how the students engaged these cognitive skills. The findings show how students used algorithmic thinking to solve maximum flow problems and then made progress towards creating a general algorithm before being introduced to the maximum-flow minimum-cut approach, which guarantees a solution.

## Introduction

Algorithmic thinking is of growing importance in mathematics education at all levels as teachers prepare students for the demands of life and work in a society characterized by the ubiquitous use of digital computational technologies and complex systems (Weintrop et al., 2016; Wing, 2006). The emerging importance of algorithmic thinking complements a more general shift towards integrating computational thinking into school curriculums to meet the current and future demands of the STEM workforce (Weintrop et al., 2016). This integration is well underway in many countries (Stephens, 2018) but is advancing at a time when research into algorithmic thinking by the mathematics education community appears to be lagging curriculum reform.

Graph theory is a topic in discrete mathematics that offers many opportunities for students to engage in algorithmic thinking (Rosenstein, 2018). In Australia, this is reflected in the revised national mathematics curriculum, which requires Year 10 students to model real-world networks with vertex-edge graphs and design, test, and refine algorithms to solve network problems (Australian Curriculum, Assessment and Reporting Authority [ACARA], 2022). In Queensland (where this study was conducted), Year 12 students studying *General Mathematics*^1^ (Queensland Curriculum & Assessment Authority [QCAA], 2019) also learn how to model a range of network problems using graphs, such as *maximum flow* problems, and use standard algorithms to solve those problems in an unplugged/paper-and-pencil environment.

Researchers have long proposed that tasks which invite students to construct an algorithm to solve a graph problem (*algorithmatizing task*) engage students in the *algorithmatizing approach* (Maurer & Ralston, 1991; Moala, 2021) or *algorithmic problem solving* (Hart, 1998). Graph theory problems are well-suited to engaging mathematics students in algorithmic problem solving because novice students can develop intuitive algorithms as an antecedent to learning the approaches used in standard algorithms (Hart, 1998). However, it remains unclear how graph algorithmatizing tasks engage students in the more contemporary construct of algorithmic thinking, and it is the purpose of this study to investigate this connection.

## Review of related literature

### Defining algorithmic thinking

The term algorithmic thinking has been used sporadically in mathematics education literature to refer to the construction of algorithms from a variety of perspectives, although the term has not been clearly defined (Knuth, 1985; Petosa, 1985; Schwank, 1993). In the 1998 NCTM yearbook about the teaching and learning of algorithms in school mathematics, Mingus and Grassl (1998) proposed the following working definition:

*Algorithmic thinking* is a method of thinking and guiding thought processes that uses step-by-step procedures, requires inputs and produces outputs, requires decisions about the quality and appropriateness of information coming in and information going out, and monitors the thought processes as a means of controlling and directing the thinking process. In essence, algorithmic thinking is simultaneously a method of thinking and a means for thinking about one’s thinking. (p. 34)

This definition emphasizes that algorithmic thinking is a method of thinking that results in the construction of a set of steps. More recently, Lockwood et al. (2016) proposed a similar working definition of algorithmic thinking based on their interviews with five mathematicians, which again emphasizes algorithmic thinking as a way of thinking that results in a set of steps: “A logical, organized way of thinking used to break down a complicated goal into a series of (ordered) steps using available tools” (p. 1591). However, these definitions are general because they do not elaborate on the proposed ways of thinking.

Stephens and Kadijevich (2020) suggest that algorithmic thinking is more than the construction of a set of steps and refer to it as a form of mathematical reasoning that is “required whenever one has to comprehend, test, improve, or design an algorithm…” (p. 2). They also propose that algorithmic thinking is comprised of decomposition, abstraction, and algorithmization (algorithm design), which is a subset of cognitive skills drawn from models of computational thinking (Shute et al., 2017).

The notion that algorithmic thinking is a set of cognitive skills required to construct, understand, or evaluate algorithms is consistent with definitions in the computer science education literature (Futschek, 2006; Korkmaz et al., 2017). The cognitive skills used to define algorithmic thinking in this field typically include understanding, analyzing, and precisely specifying a problem; developing a sequence of steps comprised of basic actions that will solve the problem; considering simple and complex, normal, and special cases of the problem; and evaluating, improving, or optimizing the efficiency of an algorithm by considering alternative approaches (Doleck et al., 2017; Futschek, 2006; Kanaki et al., 2020).

Researchers have suggested that the process of constructing an algorithm is akin to a problem-solving process, with each phase of the process requiring the use of these cognitive skills (Futschek & Moschitz, 2010; Mingus & Grassl, 1998; Ritter & Standl, 2022). One such process, proposed by Ritter and Standl (2022), involves describing, abstracting, and decomposing a problem, designing an algorithm, and then testing the algorithm. In Table 1, I use this three-stage process to structure a comparison of the cognitive skills involved in algorithmic thinking from each of the aforementioned perspectives.

**Table 1:** Perspectives on cognitive skills involved in constructing an algorithm

**Algorithmic thinking process (**Ritter & Standl, 2022**)**	**Mathematics education**	**Computer science education (**Futschek, 2006**)**	**Computational thinking (**Shute et al., 2017**)**
Describe, abstract, and decompose problem	• Break down a problem into subgoals (Maurer, 1992; Stephens & Kadijevich, 2020) • Break down a complicated goal (Lockwood et al., 2016)	• Understand and analyze a problem	• Decomposition: break down a problem or system into smaller parts
	• Abstraction (Stephens & Kadijevich, 2020)	• Specify problem precisely	• Abstraction: determine the essential components of a system
Design algorithm	• Series of ordered steps using available tools (Lockwood et al., 2016) • Algorithmization (Stephens & Kadijevich, 2020)	• Define basic actions that will solve the problem • Develop a sequence of steps comprised of basic actions	• Algorithms/algorithm design: create a sequence of steps to solve the problem
Test the solution	• Confirm or prove that the algorithm solves the problem (Maurer, 1992) • Compare algorithm with others that solve the same problem (Hart, 1998; Moala et al., 2019)	• Evaluate • Improve or consider alternative actions (Doleck et al., 2017; Ginat, 2008) • Optimize efficiency	• Debugging: detect and fix errors
	• Test and refine the algorithm using problems of the same type (Moala et al., 2019)	• Consider normal and special cases; simple and complex (Kanaki et al., 2020)	• Iteration: repeat algorithm design process

It is clear from Table 1 that there are diverse explanations of algorithmic thinking across the fields of education, and it is beyond the scope of the present paper to reconcile the differences. Nevertheless, the notion that algorithmic thinking refers to the cognitive skills (decomposition, abstraction, algorithmization, and debugging) required to construct an algorithm is shared across definitions, and this interpretation is adopted in this study.

### Algorithmatizing tasks

Evidence suggests that students’ responses to graph algorithmatizing tasks follow a process similar to that presented in Table 1. First, students make sense of a real-world problem and then construct an abstract representation using a vertex-edge graph to show the relationships between the elements of the problem (Hart & Martin, 2018). Researchers have suggested that students of all ages can construct vertex-edge graphs to represent a variety of graph problems including friendship networks (Carruthers et al., 2011), distances between locations (Ferrarello & Mammana, 2018), and compatibility of radio station locations (Hart & Martin, 2018). However, Wetzel et al. (2020) found that students struggled to construct a vertex-edge graph when they attempted to incorporate unnecessary details. Aside from this finding, there appears to be very little analysis of how students formulate vertex-edge graphs, nor how students use decomposition in the process of formulating a graph problem.

The second stage of the algorithmatizing process is to design an algorithm to solve the problem. Researchers have provided anecdotal evidence that students can design rudimentary algorithms to solve a range of graph problems including finding the shortest paths (Gibson, 2012; Wetzel et al., 2020), Eulerian cycles (Ferrarello & Mammana, 2018), and optimal assignments (Hart & Martin, 2018), but there is little analysis of how the students designed the steps in their algorithms. In contrast to these studies, Moala (2021) analyzed the instructions contained in the algorithms constructed by two groups of students for finding an optimum seating arrangement from a given graph. The analysis revealed that the students first found the optimum seating arrangement and then devised rules based on the features of their solution; a mechanism that Moala (2021) called *accounting for features of the solution*. Moala’s focus on the basic actions contained in the students’ written algorithms and the reasoning they used to devise those basic actions provides much needed insight into the cognitive skill of algorithmization. Indeed, analysis of students’ algorithmization might also focus on algorithmic concepts such as *branching* (if/then/else conditions), loops that terminate when a condition is met, and the use of variables to store intermediate results (Peel et al., 2019).

The final stage of the algorithmatizing process is testing the solution to ensure that it solves the problem and revising the algorithm if it does not. Several researchers have begun investigating how students revise their algorithms and attempt to generalize their algorithms to solve similar problems (Moala et al., 2019; Tupouniua, 2020). For example, Moala et al. (2019) asked a group of three predegree students to construct an algorithm that solves a friendship network optimization problem and then generalize their algorithm by testing it on two other friendship networks. The researchers observed that the students retained elements of their algorithms that they deemed apt for some of the networks (*local considerations*) and removed or added instructions (*patching*) when the algorithm was inapt, although the students were ultimately unsuccessful in creating a general algorithm. In response to a reanalysis of the Moala et al. (2019) study, Tupouniua (2020) concluded that the use of counterexamples is an important pedagogical tool that educators can use to help students recognize the need to revise their algorithm and then make revisions to their algorithm to accommodate the counterexample. He also stresses that counterexamples may not necessarily result in students’ designing a general algorithm, but instead help educators observe the idiosyncratic ways that students revise their algorithms by making smaller adjustments.

Researchers suggest that the objective of algorithmatizing tasks is not necessarily for students to construct general algorithms successfully (Ginat, 2008; Hart, 1998; Moala et al., 2019). Instead, incorrect algorithms provide an opportunity for students to engage in algorithm analysis (Hart, 1998), reveal to students’ their persistence with erroneous ideas (Ginat, 2008; Moala et al., 2019), or elicit divergent thinking in algorithm design (Ginat, 2008). Erroneous attempts at algorithm design may also provide the starting point for the introduction of the approaches used in standard algorithms (Hart, 1998, 2008), although there appears to be limited research into how students reconcile alternative approaches with their own algorithms.

Although researchers have long advocated for the use of graph algorithmatizing tasks, there appears to be little research into how such tasks might engage students in algorithmic thinking, in line with current curricular trends. My objective in this study was to operationalize the cognitive skills of algorithmic thinking defined above and examine how Year 12 students engage these in solving a sequence of algorithmatizing tasks that required them to solve maximum flow problems. The following research question guided my investigation:

*How do students use the cognitive skills of algorithmic thinking in graph algorithmatizing tasks?*

## Conceptual framework

To analyze how students use algorithmic thinking in response to algorithmatizing tasks, I composed a framework comprised on the cognitive skills that emerged from my review of the literature: decomposition, abstraction, algorithmization, and debugging. Table 2 contains the elaborations for each of the cognitive skills in this framework.

**Table 2:** Framework for algorithmic thinking

**Cognitive skills**	**Elaboration**
Decomposition	Break down a problem or system into smaller parts or subgoals (Shute et al., 2017; Stephens & Kadijevich, 2020)
Abstraction	Determine the essential components of a problem or system, which involves • collecting relevant information/data and disregarding irrelevant information/data (Shute et al., 2017; Wetzel et al., 2020) • building representations using the essential components that show how the problem or system works (Hart, 1998; Shute et al., 2017)
Algorithmization	Design a set of ordered steps to produce a solution or achieve a goal (Lockwood et al., 2016; Shute et al., 2017) Steps include inputs and outputs (Mingus & Grassl, 1998), basic actions (Futschek, 2006; Moala, 2021), or algorithmic concepts such as • iteration/loops (Peel et al., 2019) • variables/intermediate results (Mingus & Grassl, 1998; Peel et al., 2019)
Debugging	Test that the algorithm solves the problem or other problems of the same type, which involves • fixing errors (Moala et al., 2019; Shute et al., 2017) • considering alternative approaches (Ginat, 2008; Moala et al., 2019)

Maximum flow problems were used in this study to examine students’ engagement with algorithmic thinking. Throughout the remainder of this section, I will use the *Campground Problem* (see Appendix) to explain maximum flow problems and illustrate how the framework might be applied to analyze students’ algorithmic thinking.

### Decomposition

Decomposition is a broad term that refers to the skill of breaking down a problem or system into smaller parts so that it is more manageable (Shute et al., 2017). In maximum flow problems, decomposition involves breaking down a system into the functional elements that form the system. For example, to formulate the table for the Campground Problem, I had to break down an existing network of pipes at the school campground into the essential components, which are the *source* of water (water tank), the junctions where the water is supplied (Cabins A and B), the location where the water terminates, *sink* (Dining Hall), the pipes that connect these four locations, and the direction that water flows through each pipe.

### Abstraction

**Fig. 1:**
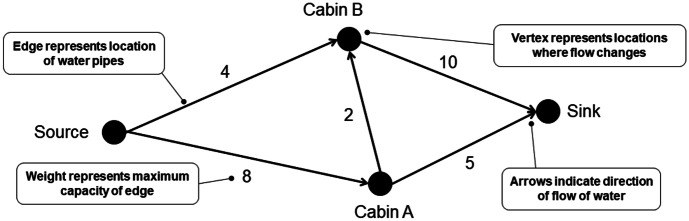
Campground network

Abstraction refers to the development of a model of a problem or system by identifying the essential components and constructing a representation of those components (Shute et al., 2017), which are used to analyze and solve problems about the system. For maximum flow problems, systems are represented by vertex-edge graphs, which are used to find the maximum flow. Figure 1 shows how an abstract model of the network of water pipes from the *Campground Problem* is represented by a vertex-edge graph. The graph shows only the information essential to describe how the system works. Irrelevant information about the system that does not affect the flow of water, such as the footpaths between the locations drawn on the map, is disregarded.

### Algorithmization

Algorithmization requires the formulation of basic actions, which are the instructions that form the ordered steps of an algorithm (Futschek, 2006) and transform inputs into outputs (Mingus & Grassl, 1998). Basic actions may incorporate mathematical operators or algorithmic concepts such as loops and variables that store intermediate results (Peel et al., 2019).

**Fig. 2:**
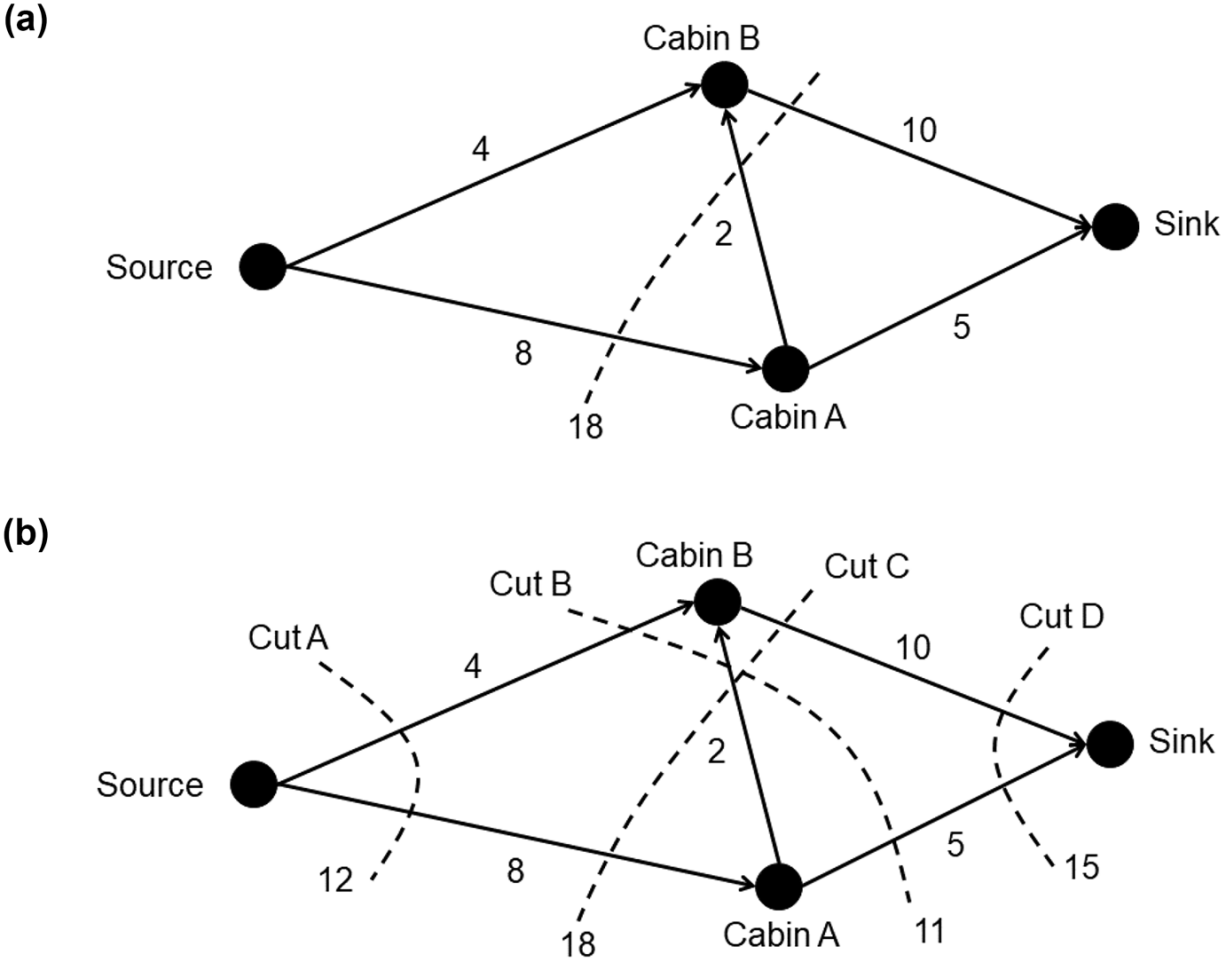
Cuts through campground network

**Fig. 3:**
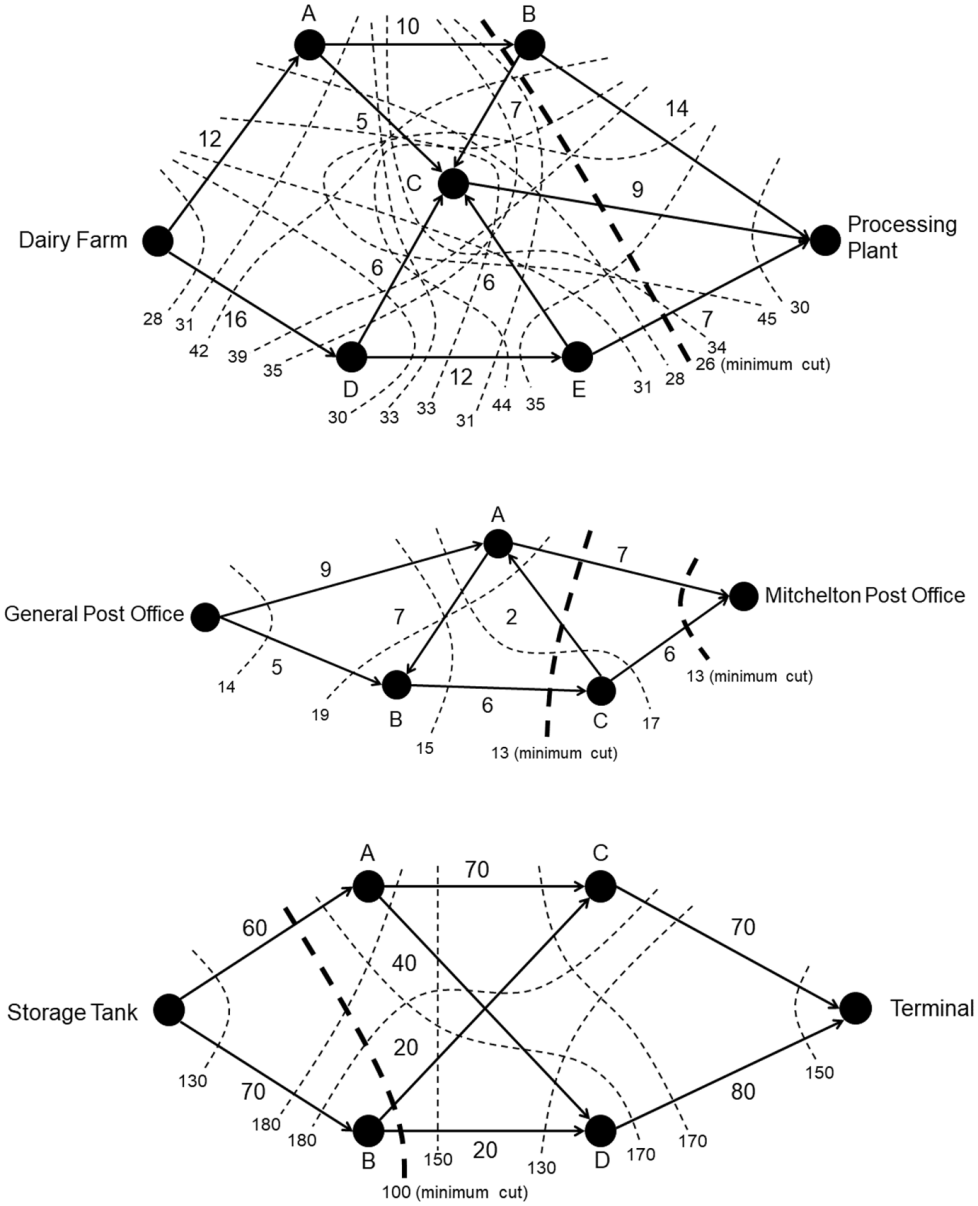
Maximum-flow minimum-cut solutions

The input for a maximum flow algorithm is the weighted graph. There are numerous algorithms for finding maximum flow^2^; however, students in Queensland must learn how to use the maximum-flow minimum-cut theorem (Queensland Curriculum and Assessment Authority, 2019), which posits that the maximum flow through a network is equal to the sum of the capacities of the minimum *cut* (Dantzig & Fulkerson, 1955). A cut is a line drawn through the graph that indicates where the source would be separated from the sink completely. Figure 2a shows a cut through the campground network because it separates the source from the sink. The capacity of the network according to this cut is 18 units/minute because 8 can flow through $Source-Cabin\;A$ and 10 through $Cabin\;B-Sink$. The capacity of the $Cabin\;A-Cabin\;B$ edge is not included in this capacity because no water can flow through this edge if the $Source-Cabin\;A$ was “cut.” However, the maximum flow through this network is 11 rather than 18. To determine this, the capacity of every cut through the network must be calculated, as shown in Fig. 2b, and the minimum capacity selected. Cut B separates the source from the sink by blocking $Source-Cabin\;B\;(4)$, $Cabin\;A-Sink\;(5)$, and $Cabin\;A-Cabin\;B\;(2)$. Each of these pipes is a bottleneck that restricts flow from the source to the sink, leaving excess capacity in the $Source-Cabin\;A$ and $Cabin\;B-Sink$ pipes. Therefore, the maximum flow of the network is the total capacity of these bottleneck pipes: $4 +5 +2 =11.$ The solutions to the *Dairy*, *Post-Office*, and *Oil-Terminal* problems (see Appendix) using this maximum-flow minimum-cut algorithm are shown in Fig. 3.

### Debugging

**Fig. 4:**
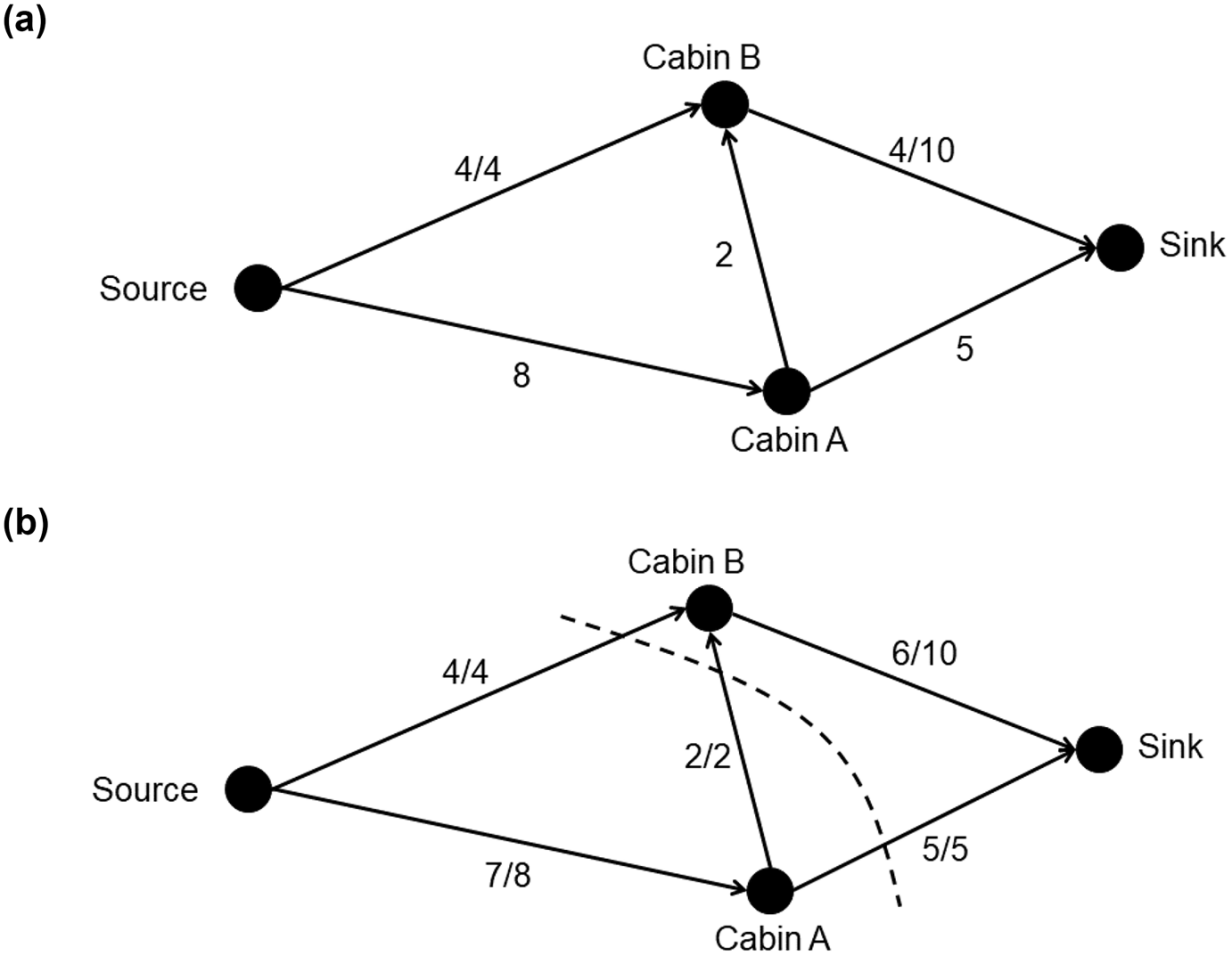
Pushing flow through campground network

The final cognitive skill in the conceptual framework is debugging, which is the ability to test and refine an algorithm so that it solves the problem successfully. Debugging might also involve considering alternative approaches if the algorithm is ineffective (Ginat, 2008; Moala et al., 2019). In the case of algorithmatizing tasks, the teacher may introduce approaches used in standard algorithms for students to consider (Hart, 1998), which is the approach taken in this study. It is unlikely that novice students would invent the minimum cut approach to finding maximum flow. Instead, they are likely to devise a basic action for finding the bottleneck in a network by examining separate paths. This basic action involves hypothetically “pushing flow” through a path until the maximum flow is found by identifying a bottleneck. For example, the maximum flow that can be pushed through the

$$Source-B-Sink$$

path in the campground network is 4 because the $Source-B$ path is a bottleneck (see Fig. 4a). This action is repeated by pushing flow through the remaining paths,

$$Source-A-Sink\;\left(5\right)$$

$$Source-A-B-Sink\;\left(2\right),$$

and summing these values,

$$4+5+2=11,$$

to find the maximum flow (see Fig. 4b). Note that the minimum cut goes through each of the bottleneck edges. This push-flow action is the basis of other standard algorithms, such as Ford-Fulkerson and Edmonds-Karp algorithms.

## Methodology

This is a generative study in that my purpose was to “generate new observation categories and new elements of a theoretical model in the form of descriptions of mental structures or processes that can explain the data” (Clements, 2000, p. 557). *Structured, task-based interviews* (Goldin, 2000) are one method used to draw inferences about students’ mathematical thinking based on their verbal and nonverbal behaviors. Hence, I conducted a series of five 45–60 min structured, task-based interviews with eight Year 12 students (40 interviews in total) to investigate how maximum flow algorithmatizing tasks engage students in algorithmic thinking.

### Participants

The eight Year 12 students (mean age = 17.5 years) were studying General Mathematics (QCAA, 2019) and in their final semester at a large, high-socioeconomic secondary school in Queensland. Table 3 contains the age and grade achieved for Semester 1 in General Mathematics, which shows that each student was above average. Each student volunteered to participate, and I obtained affirmative assent and consent from the students and their parents, respectively.

**Table 3:** Demographic information about participants

**Name (pseudonym)**	**Age at time of study**	**Grade at end of Semester 1**
Aubrey	17	A
Campbell	17	B
Frankie	17	A
Gardner	17	B
Kirby	18	B
Logan	18	A
Mackenzie	18	B
Sawyer	18	B

### Tasks

When designing the tasks for this study, I had to account for two important aspects of the curricular context. First, I considered the relevant content descriptor from the QCAA syllabus, which states that students will:

solve small-scale network flow problems including the use of the ‘maximum-flow minimum-cut’ theorem, e.g. determining the maximum volume of oil that can flow through a network of pipes from an oil storage tank to a terminal. (p. 40).

Hence, the networks designed for the study were small and the students needed to learn how to use the maximum-flow minimum cut theorem by hand, rather than the aforementioned maximum flow algorithms. Second, I considered the students’ experience in graph theory, which included learning how to construct vertex-edge graphs to represent networks and using standard algorithms to solve the shortest path, minimum spanning tree, and critical path problems. Hence, the students were familiar with the algorithmic concepts of inputs, actions, iteration/loops, and the use of variables to store intermediate and outputs.

Although the QCAA General Mathematics syllabus mandates that students apply the maximum-flow minimum-cut theorem, there is not requirement for students to design algorithmsx1. This contrasts with the senior subject *Algorithmics* (Victorian Curriculum and Assessment Authority, 2023) offered in Victoria, Australia, which focuses on graph algorithm design. Nevertheless, the approach of using algorithmatizing tasks to introduce graph algorithm problems, and as a precursor to studying standard algorithms (Hart, 1998), was familiar to the students in this study and hence was the approach adopted in this study in order to investigate the algorithmic thinking of these students.

Having considered the curricular context, I designed a sequence of five algorithmatizing tasks, which are contained in the Appendix along with the associated interview questions and contingency prompts (Goldin, 2000). Each of these tasks was designed to enable me to focus on each of the specific cognitive skills, and Table 4 contains a description of these skills in relation to each task, as well as the increasing scale of the networks in terms of number of paths and bottlenecks.

**Table 4:** Task features

**Task**	**Number of paths**	**Number of bottlenecks**	**Cognitive skill**			
			**Decomposition**	**Abstraction**	**Algorithmization**	**Debugging**
Tuckshop	1	1	• Break down system into discrete parts	• Construct graph to represent system • Quantify flows	• Devise action for identifying bottleneck	• Confirm that problem is solved • Make sense of maximum flow being minimum flow
Campground	3	3	• Break down graph into paths	• Identify relevant information and disregard irrelevant information • Construct graph from information in table (matrix)	• Devise action for determining multiple bottlenecks in a system • Devise action to calculate total maximum flow	• Confirm that the graph represents the system correcting (edges weighted; directions of flow indicated) • Consider alternative approach (e.g., brute-force)
Dairy	6	3	• Break down graph into paths	• Interpret system from given graph	• Devise action(s) for storing intermediate results	• Confirm that maximum flow is found by considering alternative solution
Post-Office	5	2	–	• Interpret system from given graph	• Devise series of ordered steps that transforms inputs into outputs • Use algorithmic concepts such as variables and loops	• Test and refine algorithm using Post-Office Problem
Oil-Terminal	4	3	–	–	• Learn how to determine capacity through a cut • Compare max-flow min-cut action with algorithm	• Consider special case

The purpose of the first two problems was to offer the students an opportunity to make sense of the goals and constraints of maximum flow problems. The Tuckshop Problem was set in a context that was familiar to the students (their school tuckshop), which would potentially enable them to make sense of a maximum flow problem along a single path, given that the tuckshop service had notoriously long waiting times. The Campground Problem context was also familiar and designed to offer an opportunity to determine relevant information from a map. The final three problems were presented with preformulated graphs so that students had an opportunity to learn how to make sense of networks that were less familiar to them (in line with the syllabus requirements).

### Data collection

**Fig. 5:**
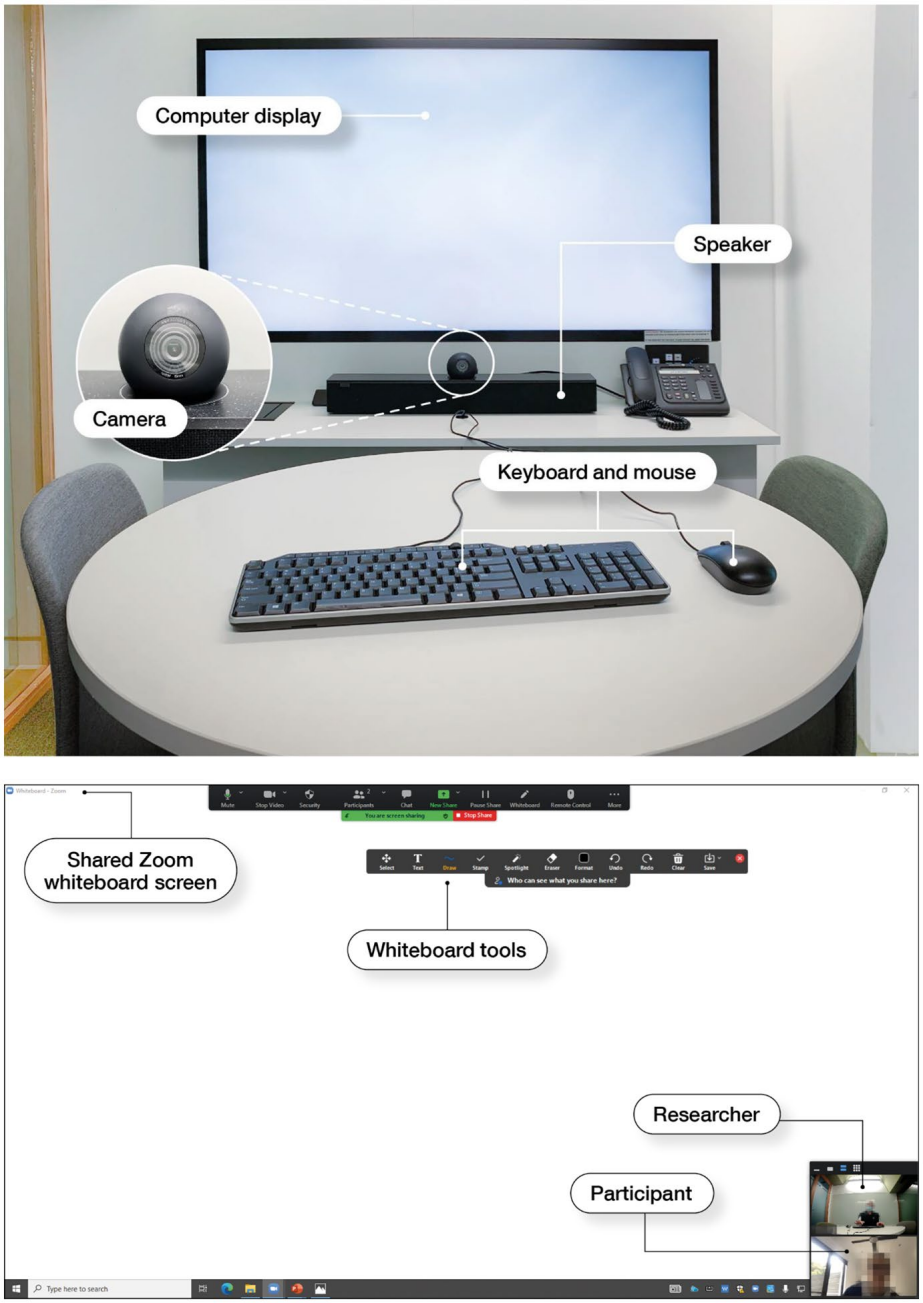
Interview room and sample Zoom screen

I conducted the interviews using Zoom because the study coincided with the COVID-19 pandemic and locally mandated restrictions prohibited students from attending the interviews in person. I accessed Zoom from a research interview room at the university, which contained a 55-inch monitor with camera and speakers that allowed me to observe the students’ mathematical behavior (see Fig. 5). The students accessed Zoom from their homes, and I could observe the students via the speaker view. During the interviews, I used Zoom’s whiteboard function to display the tasks, and the students wrote their responses onto the screen using the drawing tools. I captured screenshots of the students’ written response using the *Save Whiteboard* function in Zoom. The interviews were recorded using the application’s recording function. The data collected for the study included the following: audio and video recordings of the sessions generated by Zoom; the screenshots of all written responses produced by the students, transcriptions of the audio recordings, and students’ final algorithms.

### Data analysis

**Fig. 6:**
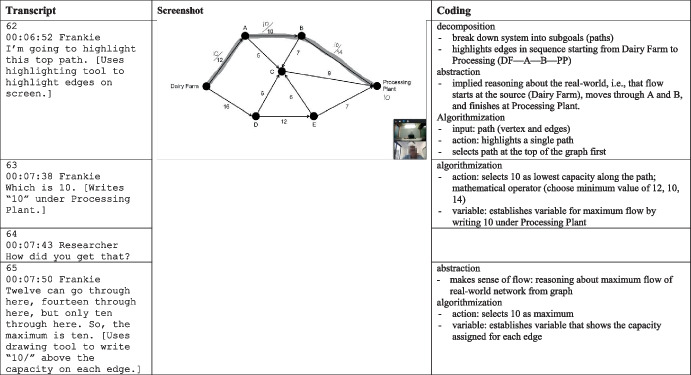
Excerpt from Frankie’s interview

I prepared the data for analysis by attaching students’ screenshots to their associated transcriptions, as shown in Fig. 6. The transcriptions included timestamp metadata that allowed me to refer to the video recordings throughout the analysis when necessary. I then analyzed the data using a two-phase, deductive-inductive approach. The first phase of the analysis was deductive as I read through each line and coded it according to the cognitive skills in the conceptual framework. For example, in Fig. 6, I coded line 62 as, “abstraction,” “decomposition,” and “algorithmization”. I then re-analyzed the data by adding the elaborations from Table 2 where applicable. For example, I coded line 62 as, “break down system into subgoals,” because the student highlighted a single path.

The second phase of the analysis was inductive. I re-read each line of the transcriptions and wrote my interpretations using memos (Corbin & Strauss, 2015). For example, my memo for line 62 in Fig. 6 was “depth-first search: highlights edges in sequence starting from Dairy Farm to Processing (DF—A—B—PP),” because this description explains how the student decomposed the network into paths. I grouped qualitatively similar types of memos together and assigned them a code. Throughout this phase of the analysis, I came to interpret these codes as *subskills* of the general cognitive skills, and I use this term in the presentation of the findings. An experienced mathematics educator reviewed my interpretations, and we resolved any conflicting interpretations through discussion. Finally, I completed a comparative analysis (Corbin & Strauss, 2015) until no new codes emerged.

**Fig. 7:**
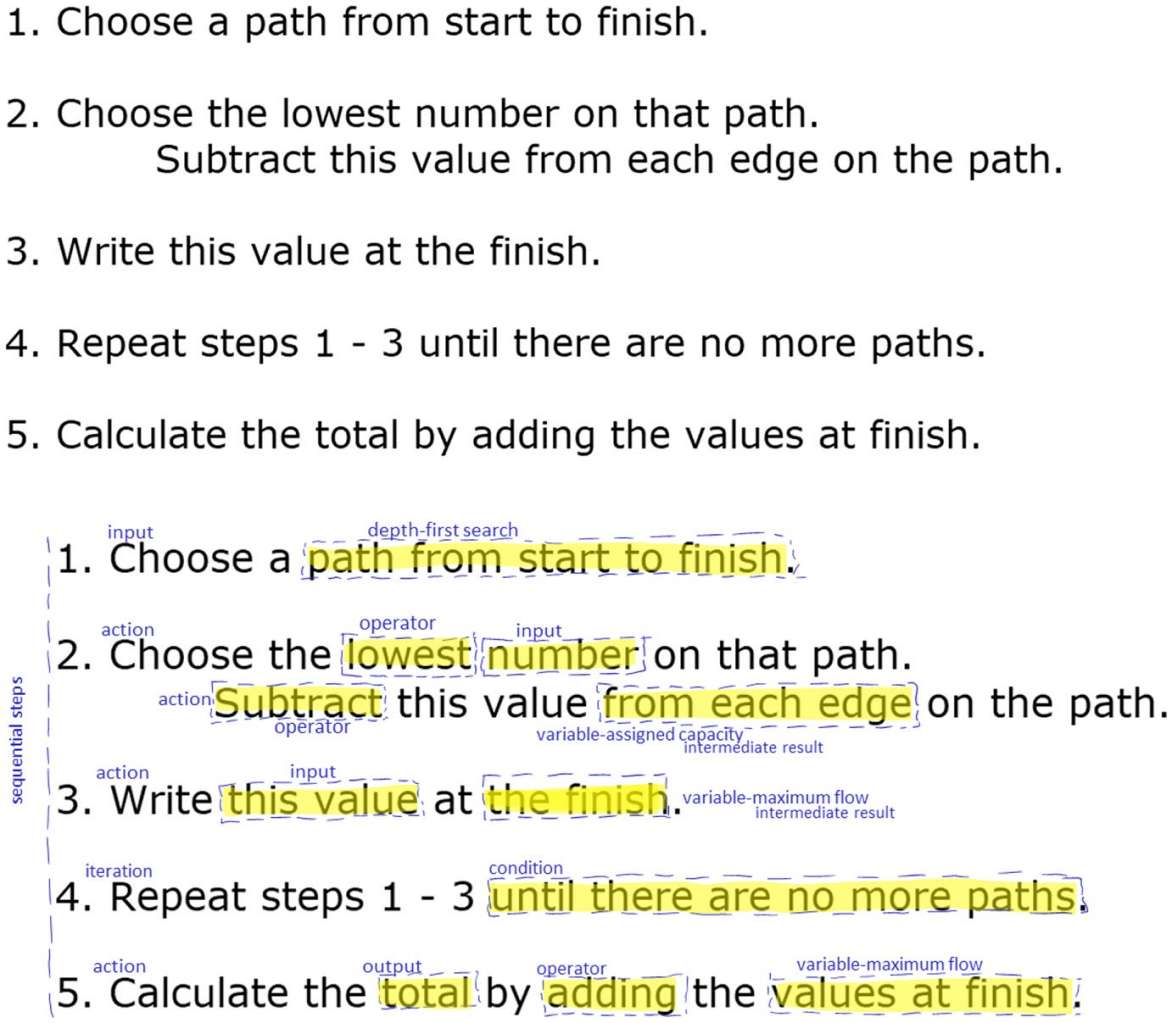
Sawyer’s algorithm before and after

After I analyzed the interview data, I turned to the students’ final algorithms. The students wrote their algorithms using plain English, as illustrated by Sawyer’s algorithm in Fig. 7. Again, I used a deductive approach to coding by using the elements of algorithms contained in the conceptual framework. First, I read the algorithms, step-by-step, and coded each step as a whole. For example, I coded Step 1 in Sawyers’ algorithm as *input*, Step 2 as *action*, and so on. Second, I read each step word-by-word, and identified phrases that indicated elements within the step. For example, I coded the phrase “this value” in Step 3 of Sawyer’s algorithm as “input” and “the finish” as “variable–maximum flow/intermediate result” because Sawyer used the lowest value on the path as the input for the variable that kept track of the total maximum flow. Finally, the previously mentioned mathematics educator reviewed my coding of all eight algorithms, and we resolved any differences through discussion.

## Findings

### Decomposition

Students used decomposition in three ways across the sequence of tasks, and I will address each of these subskills in turn.

#### Break down system into discrete activities

**Fig. 8:**
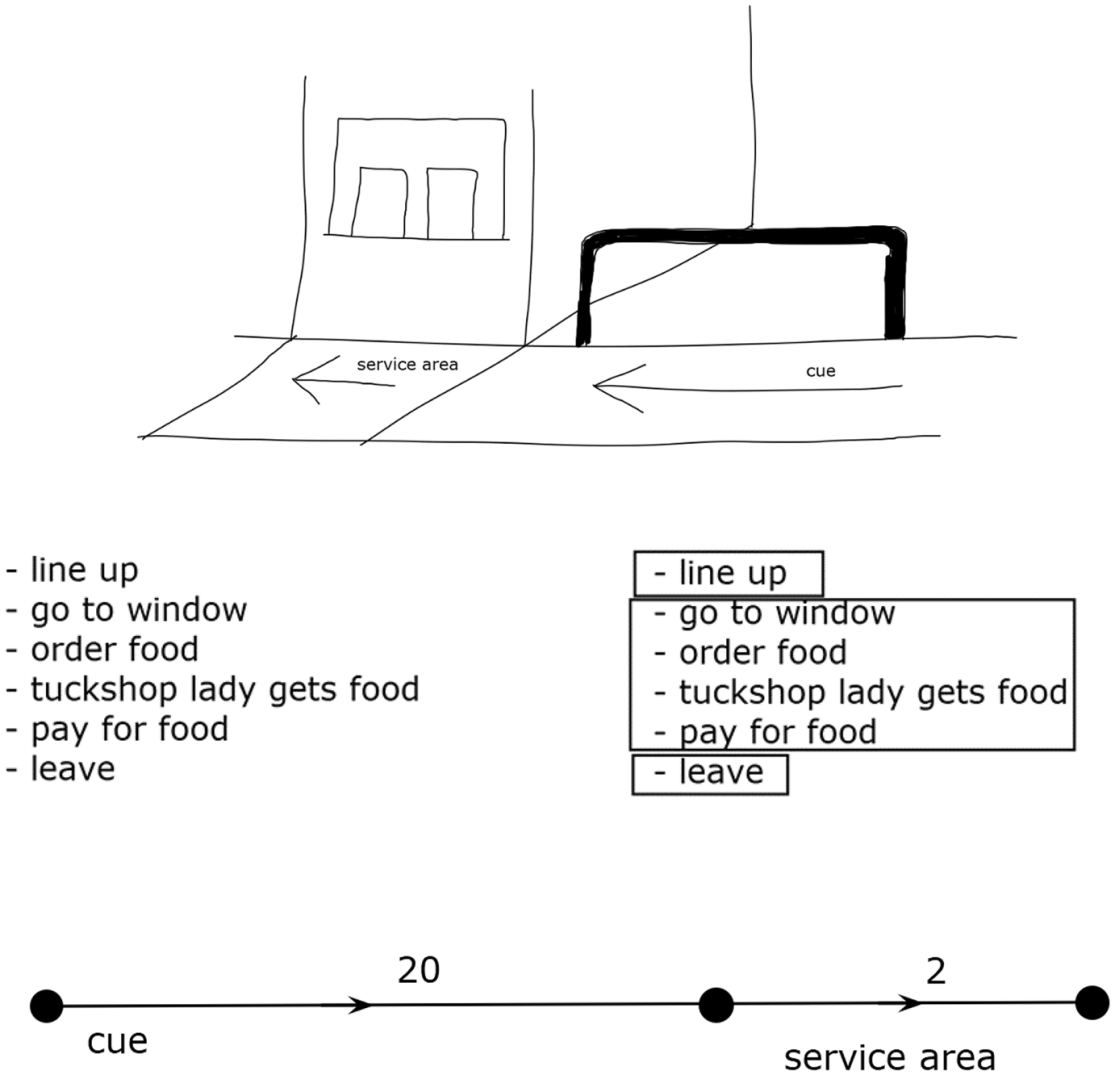
Gardner’s response to Tuckshop Problem

The students decomposed the tuckshop system into discrete activities, as illustrated by Gardner’s response (Fig. 8). Gardner first drew a diagram of the tuckshop and identified two locations in the system: the queuing and the service area. She then decomposed the process of purchasing food from the tuckshop into a list of steps and grouped these steps into discrete activities that she used to describe the flow of students through the system.

#### Break down graph into separate paths

**Fig. 9:**
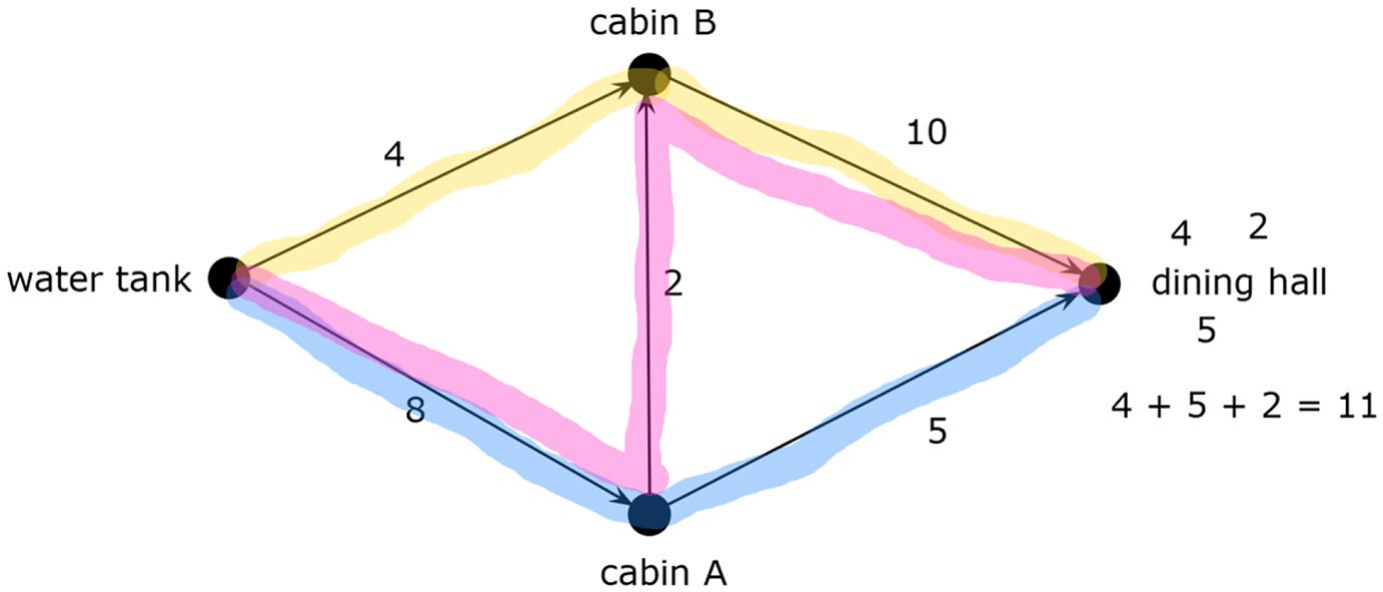
Aubrey’s decomposition of campground network

Students analyzed the graphs by identifying separate paths from source to sink. For example, Aubrey decomposed the campground network into three paths by highlighting each path in a different color (see Fig. 9). Determining the maximum flow for each path became a subgoal, the results of which she added to accomplish the overall goal of determining the maximum flow of the network.

#### Structure method or algorithm

**Fig. 10:**
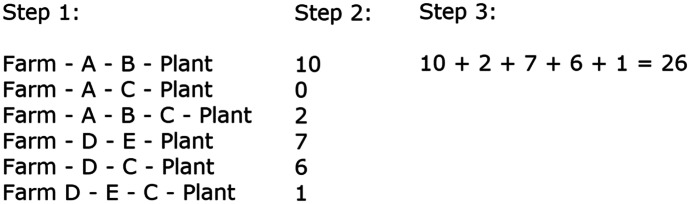
Logan’s use of decomposition in algorithm

Three students used decomposition to structure their methods for determining maximum flow. For example, Logan’s method for the Dairy Problem contained three subgoals, as illustrated in Fig. 10. The first subgoal was to find all possible paths in the network, again using decomposition. The second subgoal was to determine the maximum flow for each path, and the final subgoal was to add all the maximum flows to determine the overall maximum flow.

### Abstraction

Table 5 contains the abstraction subskills for each of the elaborations that the students used across the sequence of tasks and shows that most of these subskills emerged in response to the Tuckshop Problem. I will use Gardner’s response (Fig. 8) to illustrate each of these subskills. For *collect relevant information/data and disregard irrelevant information/data*, Gardner drew a diagram of the tuckshop and used this to visualize and make observations about the system. As outlined above, she used decomposition to form a list of discrete activities involved in ordering food from the tuckshop, but then disregarded these details by compressing them into groups of activities that related to the essential components of the system: the locations within the tuckshop. Gardner labeled the picture with the two locations (“cue” [sic] and “service area”) and drew arrows through each location to describe the flow of students. She identified that the capacity of the locations (in terms of number of students) was the relevant variable and estimated the capacities for the queuing area (20) and service area (2).

**Table 5:** Abstraction subskills

**Subskills**	**Maximum flow problems**	
*Collect relevant information/data and disregard irrelevant information/data*		
Make observations	Tuckshop Problem • Describe how system works • Identify locations within system	
	Campground Problem • Identify locations within the system from map and knowledge of campground	
Disregard information/data	Tuckshop Problem • Disregard features of the system if they do not affect flow of students [e.g., type of food and teacher on duty] Campground Problem • Disregard irrelevant information [e.g., footpath on the map of the campground]	
Identify and/or quantify variable	Tuckshop Problem • Capacity of students in each location • Estimate* capacities for queue and service area Campground Problem • Capacity of water • Determine capacities from matrix	
*Build representation using essential components that shows how system works*		
Determine type of representation	Tuckshop/Campground Problem • Vertex-edge graph	
Determine how to represent essential components	Tuckshop/Campground Problem • Edges represent locations of flow within the system • Vertices represent points where flow shifts from one location to another	
Construct representation	Tuckshop Problem/Campground Problem • Draw edges from start [source] to finish [sink], left to right, edge by edge • Position of vertex/edges roughly correspond to real-world positions, although not to scale • Arrows represent direction of flows	
*Make sense of a system*		
Read and interpret graph	All problems • Students read left-to-right, top-to-bottom • A path is comprised of a sequence of edges from source to sink • Systems are comprised of multiple paths • Not all edges will be included	
Use graph to describe real-world system	Dairy/Post-Office Problems • Trucks driving on roads from source [Farm/GPO] to sink [Processing Plant/Mitchelton PO] • Constraints caused by traffic conditions, capacities of vehicles, or activities at the Farm/GPO	
Explain how system functions	All problems • Identify constraints that give rise to maximum flow problems • Flows begin at source and end at sink • Weights indicate maximum flow through a location • Flows restricted by bottlenecks • Total flow is the quantity exiting the system	

*Originally, the Tuckshop Problem was designed so that students would collect data about the number of students that flow through each location. However, due to the COVID-19 pandemic, I removed this part of the task and students made estimates of the number of students. I stipulated that one student passes through a checkout every 30 s. All students chose values such that the number of students in the queuing area > number of students in the service area

For *build representations using essential components that shows how system works*, Gardner represented the flow of students as a vertex-edge graph, using edges to represent the two locations through which students moved, and vertices to represent the points where students entered the system, moved from the queue to the service area, and exited the system. She weighted each edge with her estimate of the capacity of students that more through each edge per minute.

For *make sense of a system*, Gardner referred to abstract representations to explain how the tuckshop system functions and identify the existence of a bottleneck in the service area that gives rise to the maximum flow problem:

**Table:** 

1	Gardner:	You line up to the tuckshop. But you’re like waiting to be served because it takes time to be served once you’re there [in the service area]
2	Researcher:	Okay
3	Gardner:	You have to ask for what you want and then they get it. Then you pay. So everyone’s waiting. Then when you leave, people move up
4	Researcher:	What is the maximum number of people that can be served per minute?
5	Gardner:	I guess it would be 2 people per minute
6	Researcher:	Why do you say that?
7	Gardner:	Um…there are two checkouts, two ladies serving…Let’s say that it takes 1 min to serve. So, its 2 students per minute

In line 1, Gardner appeared to identify the bottleneck when she argued that, “it takes time to be served once you’re there.” She then explained the cause of the bottleneck in line 3 by describing the activities that occur in the service area. Gardner also explained the flow of students through the network by first highlighting the lack of flow through the queuing area (“everyone’s waiting”) and then that the people in the queuing area “move up” after those in the service area exit the network. In lines 5 and 7, Gardner determined that the maximum flow was equal to her estimate of the number of people per minute that can be served in the service area, which is equal to the minimum capacity of the $queueing-service area$ path. Furthermore, Gardner recommended that more servers be employed to increase the capacity of the entire network.

The students also used the preformulated vertex-edge graphs to make sense of the dairy, post-office, and oil-tanker networks. Specifically, the students appeared to apply their knowledge of abstract representations to read and interpret the graphs, describe the real-world systems, and explain how the systems function. For example, in line 65 of the extract in Fig. 6, Frankie made sense of the flow of traffic when she described how much traffic could flow through each edge on a specific path, concluding that “only ten through here.” Hence, making sense of a preformulated graph appears to be a subskill of abstraction.

### Algorithmization

The analysis revealed that all eight students created lists of sequential steps comprised of elements from the methods they had developed to solve the maximum flow problems in the preceding three interviews. Table 6 contains a description of these elements as well as the problems in which the elements first emerged. Each of the eight students devised algorithms based on the push-flow approach outlined above by searching for paths with excess capacity and pushing flow through those paths.

**Table 6:** Elements of algorithms

**Element of algorithm**	**Description**	**Problem**	**Basis of action**
Input	• Capacities of edges	Tuckshop	• Familiar
	• Paths	Campground	• Familiar
Action	• Select minimum capacity for a path to determine the maximum flow for that path • Operator: < or >	Tuckshop	• Novel
	• Add the lowest capacities for each path to determine maximum flow • Operator: +	Campground	• Novel
	• Add/subtract minimum capacity to each weight on the path • Operator: + or −	Dairy/PO	• Familiar
	• Ensure there is unused capacity on a path	Campground	• Novel
Variable	• Maximum flow	Campground	• Familiar
	• Assigned capacity on each edge	Dairy	• Familiar
Intermediate result	• Record the maximum flow for a path at the sink	Campground	• Familiar
	• Update the assigned capacity at each edge	Dairy	• Familiar
Iteration	• Repeat actions for each path	Campground	• Familiar
Output	• Maximum flow	Tuckshop	• Novel
	• Bottleneck	Tuckshop	• Novel

This analysis also revealed that students combined *familiar* and *novel* actions to construct their algorithms. A familiar action refers to an instance when a student adapted an action from an algorithm learned prior to this study. For example, each student used a variable at the sink to store the maximum flow for individual paths or to store assigned/unassigned capacity on each edge (Fig. 6). A novel action, on other hand, refers to a new action that the student devised in response to the constraints of maximum flow problems. For example, each student devised an action to determine the maximum capacity for a path by selecting the lowest capacity along the path, based on their reasoning about the relationship between capacities along the paths.

**Fig. 11:**
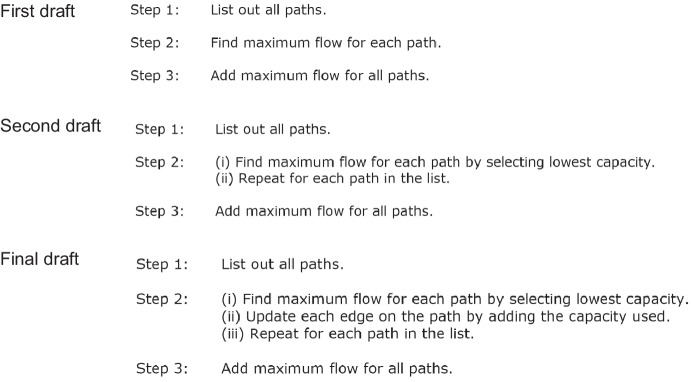
Logan’s maximum flow algorithm

All students wrote their algorithms as a set of steps but used one of two methods to compose these steps. The *solution-description* approach, used by five students, involved recounting the methods they had used to solve the preceding problems, step-by-step, and writing those steps down, as illustrated by Sawyer’s algorithm in Fig. 7. The *high-level* approach, used by the remaining three students, involved writing a high-level algorithm first, as illustrated by Logan’s first draft in Fig. 11. Logan’s first draft was comprised of a sequence of subgoals, to which she progressively added actions to reach her final algorithm.

### Debugging

After each student composed a draft algorithm, I provided the Post-Office Problem for them to test and debug their algorithms. The students tried to execute their algorithms on the post-office network as written, which exposed errors that in turn prompted revisions to their steps. Table 7 contains a summary of the types of errors that the students identified, and the types of revisions they made in response to these errors.

**Table 7:** Errors

**Errors identified**	**Revision**
Ambiguous instruction	• Add detail to step
Step delivers incorrect output	• Adjust action • Add condition
Non-terminating loop	• Add condition

An *ambiguous instruction* error refers to a step that does not contain sufficient detail to enable a user to carry it out. For example, Logan identified that Step 2 in her first draft (Fig. 11) did not contain the actions for finding the maximum flow of a path. Consequently, she added the action and iteration in the second draft. When students identified that a step delivered the incorrect output, they fixed this error by either adjusting an action or adding a condition. For example, Logan identified that Step 2 in her second draft did not deliver the correct output for GPO—A—B—C—MPO because the capacity for GPO—A had not been updated to 2. To fix this error, she added the action in Step 2 (ii) in the third draft. Some students identified that their algorithm contained a non-terminating loop. To fix such errors, students added a condition, such as Step 4 in Sawyer’s algorithm, “until there are no more paths” (Fig. 7).

#### Comparing algorithmic approaches

**Fig. 12:**
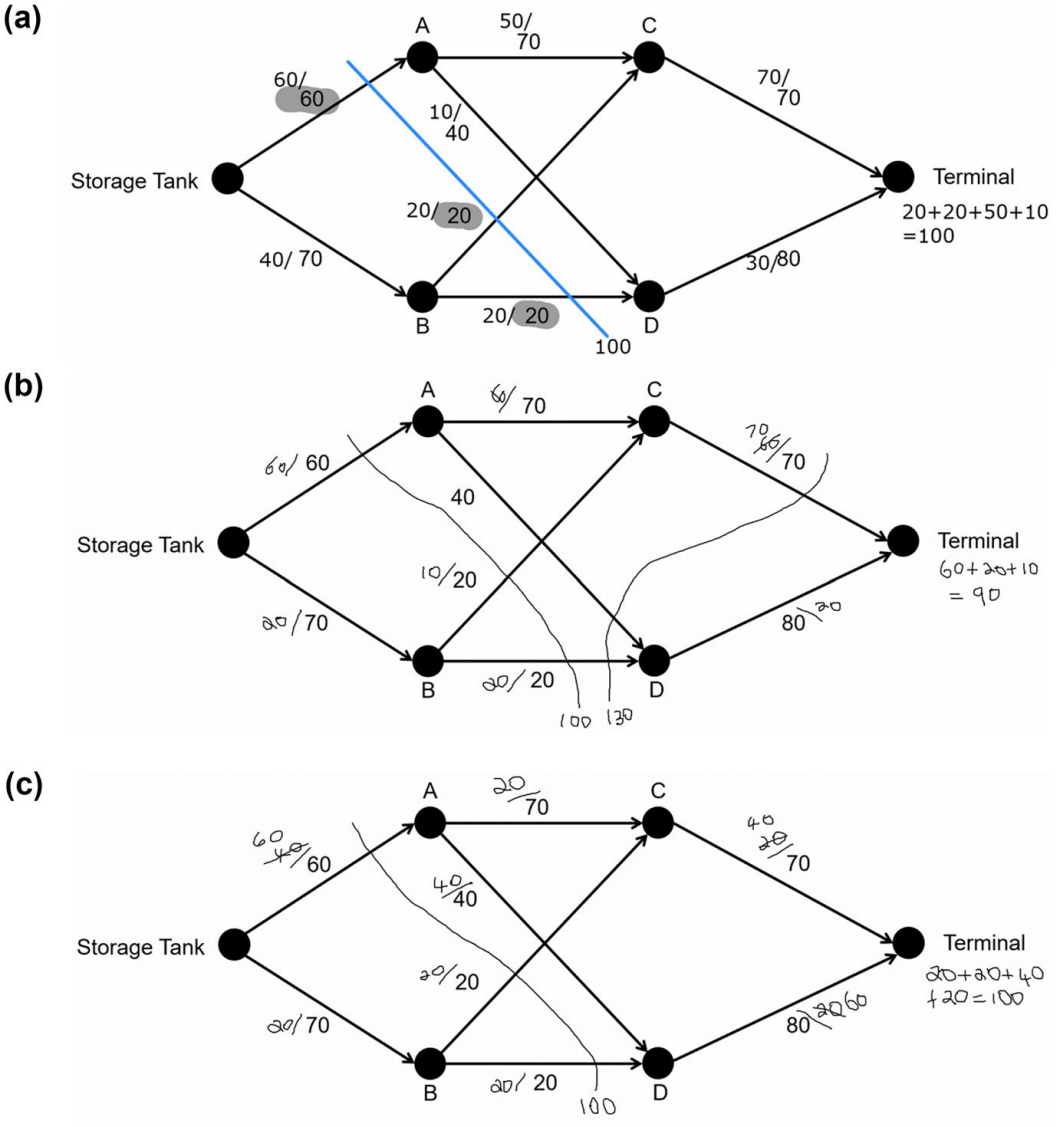
Campbell’s and Kirby’s responses to oil tanker

There were two ways that students compared the minimum-cut approach with their push-flow algorithms. Six students noticed that the minimum cut must go through all bottleneck edges and that these bottlenecks were the edges with the lowest capacity along each possible path. These students solved the Oil-Tanker problem by finding the edges with the lowest capacities on each path and drawing a minimum cut through these edges, as shown by Campbell’s response in Fig. 12a. They then used their original algorithms to verify their solution.

In contrast to this approach, Kirby and Mackenzie viewed the minimum-cut approach as a method of verifying the output generated from their own algorithms. For example, Kirby used her algorithm to find that the maximum flow through the oil-terminal network was 90 and then drew the two cuts shown in Fig. 12b, each cut through the edges with no excess capacity. Neither of these two cuts had a capacity of 90, which compelled Kirby to find which bottleneck edge had excess capacity ($B-C$). As a result, Kirby adjusted the flows so that there was no excess capacity left on the $B-C$ edge and the maximum flow equal to the minimum cut.

## Discussion

The aim of this generative study was to examine how graph algorithmatizing tasks potentially engage students in algorithmic thinking. To achieve this aim, I devised a conceptual framework for algorithmic thinking comprised of the cognitive skill decomposition, abstraction, algorithmization, and debugging and used this framework to analyze a sample of Year 12 students’ mathematical behavior as they completed a series of maximum flow tasks. The task-based interview method allowed me to identify new subskills within each of the broad cognitive skills. This helped me explain how students engaged in algorithmic thinking as they completed the algorithmatizing tasks. I will discuss each of the cognitive skills in turn, before addressing the limitations of this study.

### Decomposition

In mathematics education, decomposition and subgoals have long been taught to students as general heuristics that can be used to solve problems across the domains of mathematics (Pólya, 1945; Schoenfeld, 1985). The findings of the present study suggest that decomposition in the context of algorithmic thinking is also a cross-cutting skill because the students used it to achieve objectives in developing abstract representations, analyzing the paths in the vertex-edge graphs, and creating algorithms. Previous researchers have typically referred to decomposition in general ways and there appear to be very few examples of how students use this cognitive skill when engaging in algorithmatizing tasks (Lockwood et al., 2016; Stephens & Kadijevich, 2020). The subskills identified in the present study provided insight into how students use decomposition in algorithmatizing tasks, albeit in relation to maximum flow problems. Whether these subskills emerge in relation to other algorithmatizing tasks remains an open question.

### Abstraction

The algorithmatizing tasks used in this study revealed that students used a range of abstraction subskills to determine the essential components of the systems and related maximum flow problems. The three sets of subskills provide insights about students’ use of abstraction in algorithmatizing tasks beyond those in the existing literature. First, the students identified the essential components of the tuckshop and campground networks by making crucial observations about how the systems work, which included identifying and quantifying the relevant variable, that is, capacity (cf Hart & Martin, 2018; Wetzel et al., 2020). Previous researchers have found that students may struggle to identify the essential components of a system when they focus on contextual features (Medová et al., 2019; Wetzel et al., 2020). However, the findings of the present study highlight the importance of disregarding irrelevant information if it does not affect the system by reasoning about the real-world context.

The second set of subskills concerned how students represented the essential components of the systems using vertex-edge graphs. Previous researchers have shown that students of all ages can construct vertex-edge graphs to represent a range of networks but provide little analysis of how the students constructed these graphs (Carruthers et al., 2011; Ferrarello & Mammana, 2018; Gaio et al., 2020; Geschke et al., 2005; Hart & Martin, 2018; Wetzel et al., 2020). The findings of the present study, however, revealed that constructing vertex-edge graphs involves determining how to represent the essential components using either vertices or edges. The recordings of the shared whiteboard screens in Zoom enabled me to analyze how the students constructed the graphs, edge by edge, which revealed that students placed the vertices and edges in positions that reflected their understanding of how the system works.

The final set of abstraction subskills describes how students used vertex-edge graphs to make sense of the systems and extract information from the graphs to solve the maximum flow problems. This finding is consistent with previous studies that have provided preformulated graphs as part of algorithmatizing tasks (Moala, 2021; Moala et al., 2019). However, the findings of the present study also revealed that the process of extracting information from the graph may ultimately become an element in an algorithm created to solve the graph problem.

### Algorithmization

Although previous researchers provide anecdotal reports of students having designed graph algorithms to solve various network problems, there appears to be little analysis of the steps that students devised or how those steps emerged. The findings of this study therefore extend the limited existing research in two substantial directions. First, the students created algorithms by combining familiar and novel algorithmic concepts or actions. The students’ experience with analyzing the algorithmic concepts used in other graph algorithms, such as iteration/loops and the use of variables, supported their design. Moreover, the students’ previous experience in algorithmatizing tasks helped them recognize that novel actions needed to be devised in relation to the constraints of maximum flow problems, such as an action for selecting the minimum capacity of a single path. Devising novel actions contrasts with Moala’s (2021) mechanism of accounting for features of a solution to a problem and then “create specific rules (instructions) within the algorithm, which guarantee that the algorithm produces an object that possesses the noticed features” (p. 267). These contrasting findings, however, are most likely due to the different approaches used to design the algorithmatizing tasks; that is, Moala (2021) asked students to create an algorithm after solving a single problem whereas the students in the present study were asked to solve a sequence of problems designed to help them make sense of maximum flow problems before being asked to generate a general algorithm.

The second contribution of this study in relation to algorithmization is the finding that students composed their set of steps in two ways: either by recounting the methods they used to solve previous problems (solution-description) or by creating a high-level algorithm first and then adding further steps (high-level). Clearly, the way the set of tasks was designed most likely elicited these two approaches, and further research is needed to explore other ways that students approach algorithmization.

### Debugging

The students used two types of debugging practices—revising and adding steps—that enhanced the executability of the algorithm rather than in response to a realization that the algorithm did not necessary yield the maximum flow. This focus on the executability contrasts with the findings by Moala et al. (2019) and Tupouniua (2020) who showed how counterexamples can trigger students’ realization that their algorithms do not yield a solution and make small changes to their instructions to accommodate the counterexamples using localized considerations or patching. However, these differences might be explained by the task design used in this study, which provided students with opportunities to solve a sequence of contrasting flow network problems before designing and debugging their own algorithms. The students were able to account for the differences between (counter) example networks, such as the need to keep track of unused capacity on separate paths, in their draft algorithms before the debugging task.

In both this study and the study by Moala et al. (2019), the students made substantial progress but were unsuccessful in creating a general algorithm to solve all problems of a particular class, aside from using a brute-force approach. However, the present study extended beyond this point by introducing a divergent action (minimum cut) that guaranteed a solution, consistent with Hart’s (1998) proposition that students’ understanding of standard algorithms might be enhanced by such an approach. The students responded to the minimum cut action by either using their original algorithm to help them position the minimum cut or by using the minimum cut to verify the result of their original algorithm. Encouraging students to develop alternative or divergent approaches in response to counterexamples might be more appropriate for students enrolled in courses focused specifically on algorithm design, and further research into these students’ thinking is needed.

### Limitations

The findings about algorithmic thinking in this study are limited in five significant ways. First, the subskills are limited to maximum flow problems, and whether these subskills apply to other algorithmatizing tasks within the domain of graph theory, or in other domains of mathematics, remains an open question. Second, the reproducibility of the responses to the algorithmatizing tasks by students in other cohorts will be limited because the contexts of the Tuckshop and Campgrounds problems were familiar only to the students in this study. However, similar, simple flow problems can be devised by adapting these tasks to the networks familiar to students in other contexts. Third, the generalizability of the findings is limited because the students in this study had experience in similar graph algorithmatizing tasks and thus had developed competence in some of the subskills required by these tasks. Further investigations that involve both less and more experienced participants would enhance research into algorithmic thinking. The task-based interviews were conducted over Zoom during the COVID-19 pandemic restrictions, and more research in other task environments, particularly in classrooms, is needed. Fourth, novice students are unlikely to construct a general algorithm to solve maximum flow problems, and hence, the computability of the students’ algorithms received limited attention in this study. Instead, the students were offered an opportunity to construct their own algorithms prior to the introduction of the syllabus-mandated maximum-flow minimum-cut theorem, which guarantees a solution. Further research might examine students’ use of more divergent thinking in response to incorrect algorithms. This was not possible in this study given the time limitations imposed by the impending high-stakes examinations and COVID-19 restrictions. Finally, the subskills are limited to the sequence of tasks that I designed to support my conceptual framework for algorithmic thinking. Although I adopted an incremental approach used by other researchers (Gibson, 2012; Wetzel et al., 2020), this approach differs substantially from the designs used in more closely-related research (cf. Moala, 2021; Moala et al, 2019). These design differences limit the comparability of my study with these studies, and hence further research should focus on the differences in instructional approaches if this type of research is to inform practice.

## Appendix

### Tuckshop problem

Sam, the manager of the tuckshop, has become aware that not all students are being served during Little Lunch, with some students missing out on food entirely. He asks you to investigate the flow of students through the tuckshop so that he can implement ways of increasing the number of students served at Little Lunch.

1. Develop a model of the flow of students through the tuckshop, and determine the maximum flow of students through the tuckshop per minute.
2. Make a recommendation to Sam about how he might increase the flow of students through the tuckshop at Little Lunch.

### Campground problem

At the “School Campground,” a network of underground pipes distributes water from the water tank (source) to the two cabins and the dining hall (sink).

The volume of water that can flow through the network of pipes each minute is shown in the following table.

**Table:**

		**To**		
		**A**	**B**	**Dining hall**
**From**	**Water tank**	8	4	–
	**A**	–	2	5
	**B**	–	–	10

Determine the maximum volume of water per minute that can flow through the network.

#### Contingency prompts

1. What strategies have you used to solve other network problems?
2. Can you draw a network diagram?
3. How did you solve this problem?

### Dairy problem

Each morning, tankers transport raw milk from a dairy farm to the processing plant. The maximum volume (in KL) that can be transported along each road is shown in this network diagram.

Determine the maximum volume of raw milk that can be transported to the processing plant each morning.

#### Contingency prompt

1. How could you keep track of your intermediate results?

### Post-office problem

1. Devise an algorithm for finding the maximum flow through a network.
2. Test your algorithm by solving the *Post-Office Problem*.

This network graph shows the maximum number of postal deliveries between the *General Post Office* and the *Mitchelton Post Office* each day. Vertices A, B, and C represent other depots in this region.

Determine the maximum number of deliveries that can be made between the General Post Office and the Mitchelton Post Office each day.

### Oil-terminal problem

1. Use the Maximum-Flow Minimum-Cut Theorem to find the maximum flow of the Diary and Post-Office networks
2. The following graph shows the volume of oil that can flow through a network of pipes from a storage tank to the terminal where it is discharged onto ships. Determine the maximum volume of oil that can flow through the network and show the minimum cut on the graph.
3. Compare the maximum-flow minimum-cut approach with your algorithm.

#### Contingency prompt

If a student uses brute-force algorithm to find minimum cut, how could you verify your solution?

## Footnotes

1 General Mathematics is a non-calculus course offered to senior students whose intended future study or work does not require a knowledge of calculus and is based on the ACARA Senior Secondary curriculum.

2 such as the *Ford-Fulkerson* (Ford & Fulkerson, 1956) and *Edmonds-Karp* (Edmonds & Karp, 1972) algorithms. In fact, research into algorithms to solve maximum flow problems continues, and Chen et al. (2022) recently presented a new algorithm that reduces the processing time substantially.
